# Motif-independent *de novo* detection of secondary metabolite gene clusters—toward identification from filamentous fungi

**DOI:** 10.3389/fmicb.2015.00371

**Published:** 2015-05-05

**Authors:** Myco Umemura, Hideaki Koike, Masayuki Machida

**Affiliations:** Bioproduction Research Institute, National Institute of Advanced Industrial Science and TechnologyTsukuba/Hokkaido, Japan

**Keywords:** secondary metabolism, bioinformatics tool, filamentous fungi, gene expression, non-syntenic block

## Abstract

Secondary metabolites are produced mostly by clustered genes that are essential to their biosynthesis. The transcriptional expression of these genes is often cooperatively regulated by a transcription factor located inside or close to a cluster. Most of the secondary metabolism biosynthesis (SMB) gene clusters identified to date contain so-called core genes with distinctive sequence features, such as polyketide synthase (PKS) and non-ribosomal peptide synthetase (NRPS). Recent efforts in sequencing fungal genomes have revealed far more SMB gene clusters than expected based on the number of core genes in the genomes. Several bioinformatics tools have been developed to survey SMB gene clusters using the sequence motif information of the core genes, including SMURF and antiSMASH. More recently, accompanied by the development of sequencing techniques allowing to obtain large-scale genomic and transcriptomic data, motif-independent prediction methods of SMB gene clusters, including MIDDAS-M, have been developed. Most these methods detect the clusters in which the genes are cooperatively regulated at transcriptional levels, thus allowing the identification of novel SMB gene clusters regardless of the presence of the core genes. Another type of the method, MIPS-CG, uses the characteristics of SMB genes, which are highly enriched in non-syntenic blocks (NSBs), enabling the prediction even without transcriptome data although the results have not been evaluated in detail. Considering that large portion of SMB gene clusters might be sufficiently expressed only in limited uncommon conditions, it seems that prediction of SMB gene clusters by bioinformatics and successive experimental validation is an only way to efficiently uncover hidden SMB gene clusters. Here, we describe and discuss possible novel approaches for the determination of SMB gene clusters that have not been identified using conventional methods.

## Introduction

Filamentous fungi produce a wide variety of secondary metabolites, some of which have industrial value, including potential for medical use. Recently developed large-scale screening technology using high-throughput robotics (High-Throughput Screening: HTS), typically by pharmaceutical companies, has dramatically accelerated the possibility of discovering novel compounds from microorganisms compared to the enormous efforts made before the introduction of HTS. Compared to chemically synthesized compounds, secondary metabolites or natural compounds have larger molecular weights, more complex structures, and often chirality. These characteristics are thought to result from the multiple enzymatic reactions involved in the synthesis of these compounds. A long history of studying secondary metabolism has revealed the involvement of enzymes with remarkable characteristics, such as polyketide synthase (PKS) and non-ribosomal peptide synthetase (NRPS), which play major roles in synthesizing the backbone structure of metabolites. Enzymes such as cytochrome P450 monooxygenase, dehydrogenases, and methyltransferases are often involved in secondary metabolite biosynthesis (SMB) as well, although they are not specific to secondary metabolism. The genes encoding these enzymes have their own sequence characteristics and/or sequence motifs, which suggested that the genes are involved in secondary metabolism based on sequence similarity.

Revolutionary developments in sequencing technology have allowed us to sequence the genomes of fungal species in a relatively short period with reasonable cost and quality. Currently, the genome sequences of more than 1000 fungi have been deposited in the NCBI genome database as of March 2015 (NCBI genome database, http://www.ncbi.nlm.nih.gov/genome/browse/). The early days of fungal genomic analysis led to the discovery of genes containing motifs found in PKS, NRPS, and other enzymes known for SMB in the genomes of filamentous fungi such as *Neurospora crassa* (Galagan et al., 2003) and *Magnaporthe grisea* (Dean et al., 2005). The numbers of genes encoding PKS and NRPS in the individual genomes of eight *Aspergillus* species range from 17 to 35 and from 14 to 24, respectively (Rank et al., 2010), values much higher than expected before genome sequencing. Several bioinformatics tools have been developed to predict secondary metabolic genes. One major type of the tools first developed depends on the presence of domains typically existing in PKS, NRPS, and other known genes catalyzing the synthesis of secondary metabolites such as SMURF (Khaldi et al., 2010) and antiSMASH (Medema et al., 2011). In addition to these motif-dependent tools, motif-independent tools has been reported for detecting co-regulated gene clusters using transcriptome data. These tools take advantage of large-scale transcriptomic data, rapidly growing by the use of next generation sequencing technologies.

In this review, we describe the current development of bioinformatics tools for comprehensive detection of fungal SMB gene clusters, particularly those without the use of known motifs. Of them, we take a closer look at MIDDAS-M, which successfully discovered the ustiloxin B biosynthesis gene cluster encoding a novel pathway, Ribosomal Peptide Synthetic (RiPS) pathway in fungi, for the biosynthesis of a peptide compound in a manner different from NRPS.

## Characteristic localization of secondary metabolism genes on chromosomes

The sequencing of three *Aspergillus* species provided the first good opportunity for detailed genome comparison between closely related species of filamentous fungi (Galagan et al., 2005; Machida et al., 2005; Nierman et al., 2005). Syntenic analysis between the genomes of *A. oryzae* and *Aspergillus nidulans* or *Aspergillus fumigatus*, revealed that non-syntenic blocks (NSBs) were distributed in a mosaic manner throughout the *A. oryzae* genome. In contrast to syntenic blocks (SBs), which harbored genes involved in basic cellular functions, NSBs, which occupy approximately 25% of the *A. oryzae* genome, harbored a large proportion of genes that were predicted to belong to secondary metabolism and secretory hydrolases.

**Figure 1:**
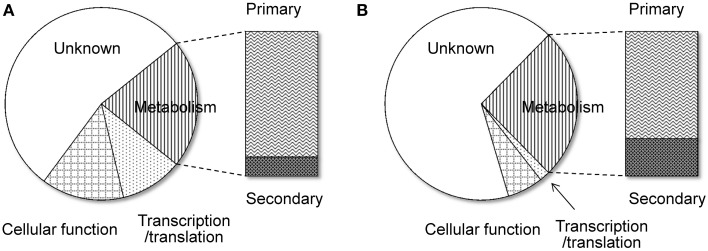
**Functional difference between the genes on SBs and NSBs**. Genes in the *A. oryzae* genome were functionally classified by searching for them in the COG/KOG database (Tatusov et al., 2003) using BLASTP. **(A)** SBs, **(B)** NSBs.

Another remarkable feature of the NSB genes is that a significantly larger proportion of the genes are functionally unknown compared to the SB genes (Tamano et al., 2008; Umemura et al., 2012). Figure 1 shows functional analyses of the *A. oryzae* genes localized to SBs and NSBs. Analysis using the Cluster of Orthologous Group (COG) and Eukaryotic Orthologous Group (KOG) (Tatusov et al., 2003) reveals that genes of unknown function comprise 67% of NSB genes, a significantly larger proportion than the 54% unknown function genes in SBs. In spite of a similar proportion of metabolism genes in SBs and NSBs, NSBs contain a larger proportion of genes involved in secondary metabolism and genes encoding secretory hydrolases and transporters than SBs. Table 1 shows the localization of the PKSs and NRPSs identified in the *A. oryzae* genome. Although NSBs occupy only approximately 25% of the entire genome, 64% of PKS/NRPS genes are located in NSBs, a 2.5-fold enrichment in NSBs. Similar genomic islands were found in *A. fumigatus* (Perrin et al., 2007; Fedorova et al., 2008), *Magnaporthe oryzae* (Rehmeyer et al., 2006; Thon et al., 2006) suggesting common feature for genomics of filamentous fungi. Although most of the software tools to predict SMB gene clusters do not use the close relationship between SMB genes and NSBs described above, the information could play an important role for the development and evaluation of the software tools.

**Table 1:** **Localization of the PKS/NRPS genes of *A. oryzae***.

**Function**	Gene ID^*1^	***E*-value**	Top hit by BLASTP search^*2^
**LOCATED ON SBs**			
PKS	AO090026000001	1.00E4−161	Polyketide synthase-nonribosomal peptide synthetase [*Aspergillus oryzae*]
PKS	AO090206000074	0.00E+00	Polyketide synthase PKSL2 [*Aspergillus parasiticus*]
PKS	AO090701000530	0.00E+00	PKS-like enzyme, putative [*Aspergillus flavus* NRRL3357]
NRPS	AO090003001097	0.00E+00	L-aminoadipate-semialdehyde dehydrogenase large subunit [*Aspergillus terreus* NIH2624]
NRPS	AO090103000167	0.00E+00	Non-ribosomal siderophore peptide synthase Sid2 [*Aspergillus flavus* NRRL3357]
NRPS	AO090005001026	0.00E+00	AMP-dependent ligase, putative [*Aspergillus flavus* NRRL3357]
NRPS	AO090001000516	0.00E+00	NRPS-like enzyme, putative [*Aspergillus flavus* NRRL3357]
NRPS	AO090003000945	0.00E+00	NRPS-like enzyme, putative [*Aspergillus flavus* NRRL3357]
**LOCATED ON NSBs**			
PKS	AO090102000545	0.00E+00	Polyketide synthetase PksP [*Aspergillus flavus* NRRL3357]
PKS	AO090113000209	0.00E+00	Polyketide synthase, putative [*Aspergillus flavus* NRRL3357]
PKS	AO090010000048	0.00E+00	Polyketide synthase, putative [Aspergillus flavus NRRL3357]
PKS	AO090005000961	0.00E+00	Polyketide synthase, putative [*Aspergillus flavus* NRRL3357]
PKS	AO090010000404	0.00E+00	Polyketide synthase, putative [*Aspergillus flavus* NRRL3357]
PKS	AO090701000826	0.00E+00	Polyketide synthase, putative [*Aspergillus flavus* NRRL3357]
PKS	AO090009000131	0.00E+00	Polyketide synthase [*Botryotinia fuckeliana*]
NRPS	AO090103000223	0.00E+00	Non-ribosomal peptide synthase, putative [*Aspergillus flavus* NRRL3357]
NRPS	AO090026000585	1.00E−157	Non-ribosomal peptide synthase [*Aspergillus fumigatus* Af293]
NRPS	AO090026000378	0.00E+00	Non-ribosomal peptide synthase, putative [*Aspergillus flavus* NRRL3357]
NRPS	AO090102000338	0.00E+00	Non-ribosomal peptide synthase, putative [*Aspergillus flavus* NRRL3357]
NRPS	AO090010000498	0.00E+00	NRPS-like enzyme, putative [*Aspergillus flavus* NRRL3357]
NRPS	AO090020000240	0.00E+00	NRPS-like enzyme, putative [*Aspergillus flavus* NRRL3357]
NRPS	AO090003001545	0.00E+00	NRPS-like enzyme, putative [*Aspergillus flavus* NRRL3357]

*1 *The list of PKS and NRPS genes was obtained from Umemura et al. (2013b)*.

*2 *The NCBI nr-aa database was used, but the top hits from the A. oryzae genome were removed*.

## Detection methods for SMB gene clusters using transcriptome data

One conventional way to survey SMB genes from fungal genome sequences is to use known sequence motifs of enzymes involved in biosynthesis of a metabolic backbone such as PKS, NRPS, or dimethylallyl tryptophan synthases (DMATs) (Fedorova et al., 2012). The genes encoding these enzymes are called “core genes” or “backbone genes.” SMURF (Khaldi et al., 2010), antiSMASH (Medema et al., 2011; Blin et al., 2013), ClustScan (Starcevic et al., 2008), and CLUSEAN (Weber et al., 2009) adopt this strategy and predict gene clusters typically composed of approximately 10–30 genes including the SMB core genes. The central algorithm used in these tools is hidden Markov model (HMM), in addition to BLAST homology search algorithm (Altschul et al., 1990; Li et al., 2009), which depend certain sequence information of domains and genes. The methods are summarized in Table 2.

**Table 2:** **Detection methods for secondary metabolite biosynthetic gene clusters**.

**Type**	**Method**	**Algorithm^a^**	**References**	**Fungal strains tested in reference**
Motif-dependent	SMURF	HMM	Khaldi et al., 2010	*A. flavus, A. oryzae, A. nidulans, A. niger, A. fumigatus, A. terreus, A. clavatus, N. fischeri*, et al.
	antiSMASH	profile HMM	Medema et al., 2011	*A. fumigatus*
	antiSMASH 2.0	profile HMM	Blin et al., 2013	–
	ClustScan	HMM	Starcevic et al., 2008	–
	CLUSEAN	Blast, HMM	Weber et al., 2009	–
	NP.searcher	Blast	Li et al., 2009	–
Motif-independent	Andersen's	Sliding window with coregulation coefficient	Andersen et al., 2013	*A. nidulans*
	Gibbons's	Sliding window with coregulation probability	Gibbons et al., 2012	*A. fumigatus*
	MIDDAS-M	Sliding window with deviation from standard distribution	Umemura et al., 2013a	*A. flavus, A. oryzae, F. verticillioides*
	MIPS-CG	Comparative genomics	Takeda et al., 2014	*A. flavus, A. oryzae, A. nidulans, A. fumigatus, A. terreus, F. verticillioides, F. graminearum, F. oxysporum, C. globosum, M. grisea*

a *HMM: hidden Markov model*.

In addition to these tools using motif information of the core genes for the prediction of SMB gene clusters, a few tools are recently developed using transcriptome data to detect gene clusters in which the genes are cooperatively regulated. Andersen et al. defined the method to score the co-regulation of adjacent three genes. Using transcriptome data from 44 samples including four strains of *A. nidulans*, four different growth media, and five different carbon sources, they precisely determined the boundaries of 70 SMB gene clusters containing the core genes (Andersen et al., 2013). Gibbons et al. performed sliding window analysis to evaluate co-regulated gene clusters in *A. fumigatus*, and successfully detected 27 gene clusters upregulated under biofilm-like state, among which seven clusters are considered to be SMB ones as judged from existence of the core genes (Gibbons et al., 2012). Dhingra et al. adopted the method by Gibbons et al. to detect gene clusters regulated by the global transcription factor, *veA*, in *A. fumigatus* and successfully detected several potential SMB gene clusters (Table 2) (Dhingra et al., 2013). These methods are mainly used in combination with information of SMB core genes, to analyze fungal SMB gene clusters.

Transcriptomic analysis can be one of the best ways to identify the corresponding SMB genes of a fungus that produces a metabolite of interest. Secondary metabolism is “not essential to growth” and is activated only when necessary; transcriptional regulation plays a crucial role in this activation. Many SMB genes are activated after a logarithmic growth phase. In addition, various factors that affect the induction of secondary metabolism, such as temperature, carbon/nitrogen ratio, and medium composition, can potentially make an inducible condition very specific to each secondary metabolism (Brakhage, 2013). Therefore, analyzing the transcription profiles of producing and non-producing conditions and successively comparing these profiles should be an effective way to identify SMB gene clusters. Based on this idea, another motif-independent method, MIDDAS-M, was developed to detect gene clusters, whose component genes are co-regulated (Umemura et al., 2013a). This method uses induction ratios of whole genes under compound producing over non-producing conditions.

Most fungal SMB gene clusters already known to date include the core genes, but some gene clusters do not contain any of the SMB core genes. Recently, the genes responsible for kojic acid (KA) biosynthesis were identified from the *A. oryzae* genome (Terabayashi et al., 2010). The KA biosynthesis gene cluster consists of three genes, which are frequently found in SMB gene clusters, namely a Zn_2_-Cys_6_ (C6) fungal-type transcription factor, a major facilitator superfamily (MFS) transporter, and an oxidoreductase genes. However, none of the three genes are the core genes. This example clearly illustrates the importance of developing new methods for predicting genes involved in a novel SMB pathways.

## Identification of a kojic acid biosynthesis gene cluster using the transcriptome

A gene cluster for the biosynthesis of KA is one of the typical examples without the core genes. Small cluster size consisting of only three genes makes its detection more difficult as compared to most of the known SMB gene clusters consisting of 10–30 genes in general. KA was first identified in 1907 from a *koji*-culture, a solid-state culture of steamed rice inoculated with *A. oryzae* (Saito, 1907). However, despite the importance of KA, especially in the fermentation industry, the genes responsible for its production were only first identified in 2010 (Terabayashi et al., 2010). Biosynthesis pathway analysis of KA using an isotope tracer technique suggested the direct conversion of glucose to KA by no more than two or three enzymes (Arnstein and Bentley, 1953a,b,c), indicating that no SMB genes with typical motifs such as PKS or NRPS were involved.

To identify the genes responsible for KA biosynthesis, the KA-producing/KA-non-producing expression profiles of three pairs of conditions were selected for analysis: (i) without nitrate/with nitrate, (ii) 4th day/2nd day after cultivation, (iii) 7th day/4th day after cultivation (Table 3). Despite significant induction of KA production in all three pairs of conditions, no genes were found that were induced in more than two of the three pairs of conditions. Thus, the first two genes, AO090113000136 (*kojA*, oxidoreductase) and AO090113000138 (*kojT*, transporter), which were located very close to each other, were originally identified by one-by-one disruption approach referring to the transcriptome data. An additional gene, AO090113000137 (*kojR*, transcription factor) was found in between the two genes above.

**Table 3:** **Induction ratio rankings for the KA biosynthesis genes**.

**Producing/Non-producing**	**Index**	**AO090113000136 (*kojA*)**	**AO090113000137 (*kojR*)**	**AO090113000138 (*kojT*)**	**Cluster**
7th day/4th day	*t*-value	5	22	2	1
	*m*×*a*	1	14	2	1
	*m*-value	12	56	9	1
	*a*-value	51	1575	554	46
4th day/2nd day	*m*-value	1352	2216	1413	1126
Nitrate(−)/Nitrate(+)	*m*-value	2022	1185	1074	1103

In order to address most important factors for the detection of a co-regulated gene cluster only from gene expression profiles, several values obtained from simple calculation and statistics are compared using the KA gene cluster as a model case (Table 3). For the transcriptome data from the 7th day/4th day pair, the gene expression induction ratios (*m*-values) of *kojA, kojR*, and *kojT* rank relatively high: 12, 56, and 9, respectively. However, choosing the correct genes from these *m*-values is difficult, as the correct genes are not ranked first or very close to first. Calculating *t*-values using the statistical *t*-test significantly improved the selection power, as the *kojA, kojR*, and *kojT* rankings jump to 5, 22, and 2 from 12, 56, and 9, respectively. Another measuring index, *m*×*a*, which is calculated by multiplying the *m*-value by the average expression level (*a*-value) based on high productivity of KA, is also effective for selecting the correct genes: the rankings of the three genes are 1, 14, and 2, respectively. However, the *m*-values and other indexes for the three genes rank from 1000 to 2000 for the 4th day/2nd day condition pair and the nitrate(−)/nitrate(+) pair. As KA production significantly increases for all three pairwise conditions, it is fairly difficult to select suitable conditions (in this case, the 7th day/4th day pair) to generate a useful transcriptome dataset for the identification of genes responsible for KA production.

To resolve this difficulty, an approach to select SMB genes based on the general characteristic of cooperative induction when they are producing secondary metabolites were developed. These genes, including genes related to well-known metabolites such as aflatoxin, sterigmatocystins, melanin, and trichothecenes, are clustered and co-regulated by a pathway-specific transcription factor (Keller and Hohn, 1997). In contrast, eukaryotic genes are generally not clustered in the genome, regardless of relationship of their functions. Therefore, averaging the *m*-values of the genes in a typical genomic region should yield a value close to zero, while the average *m*-value of the genes in an SMB gene cluster would yield a significantly larger value.

To test the above hypothesis, the average *m*-values are calculated for all possible virtual gene clusters containing three genes by moving a three-gene window function one gene at a time through the annotated genes of the *A. oryzae* genome. As expected, the average *t*-value, *m*-value and *a*-value for the KA-biosynthesis gene cluster are ranked 1st in the lists using the 7th day/4th day cultivation condition pair (Table 3). These results suggest that fluctuation noise in the gene expression values can be suppressed by averaging, particularly when the cluster contains a large number of genes. Noise suppression by averaging is the fundamental concept behind MIDDAS-M, which detects SMB gene clusters based on transcriptome data. However, the final form of the algorithm (described below) uses the summation, not the average, to evaluate cluster scores and uses other statistical tricks to improve detection sensitivity.

## Mechanism of MIDDAS-M

**Figure 2:**
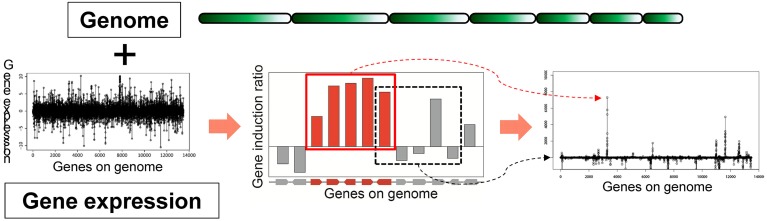
**Principles of MIDDAS-M**. MIDDAS-M uses both genomic information, in which gene positions are annotated, and transcriptome data. When adjacent genes are co-expressed, as is the case with secondary metabolic genes, the sum of their induction ratios or expression values becomes large, whereas other summed values approach the average for all gene values.

**Figure 3:**
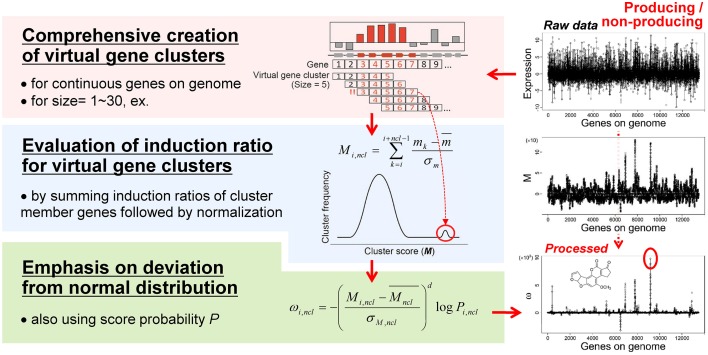
**The MIDDAS-M algorithm**. After creating all possible virtual gene clusters for a genome sequence, the induction ratios (*M*) of the virtual gene clusters are evaluated by summing the induction ratios of the genes in each cluster. The SMB gene cluster candidates should have large *M*-values that deviate from the normal distribution, which is emphasized by the statistical evaluation.

Based on the idea of evaluating the expression value per gene cluster to detect functional SMB genes, we have developed a novel method to detect SMB genes that without using motif information from the SMB core genes. We named this method MIDDAS-M, which stands for motif-independent *de* novo detection algorithm for SMB gene clusters. As shown in Figure 2, the foundation of this method is the creation of a comprehensive list of virtual gene clusters (VCs) in a genome sequence, followed by the summation of the expression ratios of the genes in each VC. Using this method, the VCs containing genes that are co-expressed have large values, whereas other VCs have values close to the average. In other words, the gene induction ratios are distributed normally in a symmetric bell-shaped curve, but VCs that are candidate SMB clusters exhibit large absolute values that deviate from the symmetric normal distribution curve (Figure 3, middle). For a given cluster size *ncl*, this procedure can be described using the following equation:

(1) $$M_{i,\;ncl}=\sum_{k=i}^{i+ncl-1}\frac{m_{k}-\bar{m}}{\sigma_{m}}$$

where *m_k_* is the induction ratio or gene expression level of gene *k*, and m and σ*_m_* are the mean and the standard deviation of all *m*-values, respectively. This equation normalizes the gene induction ratios via Z-score transformation, which makes the average zero and the standard deviation 1. After normalization, the top of the bell-shaped distribution centers on the *y*-axis (*x* = 0). Note that the *M* scores become largely negative when the expression value is the induction ratio of secondary metabolite non-producing vs. producing conditions.

**Figure 4:**
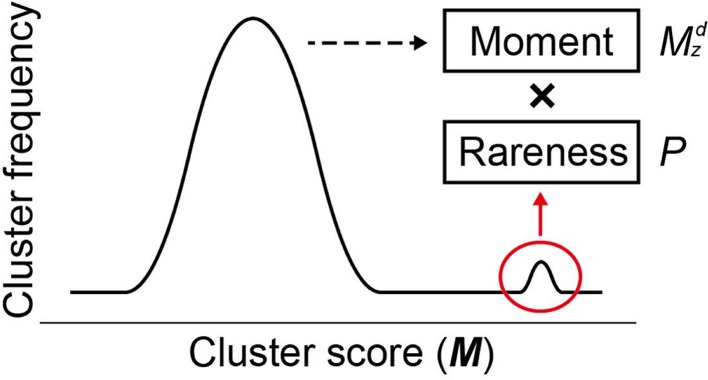
**Two factors for high sensitivity of MIDDAS-M**. Based on the principle of MIDDAS-M, the algorithm emphasizes the *M*-values of the SMB gene cluster candidates using two factors: moment and rareness. The deviation of the *M*-values from the normal distribution is amplified by the moment, and the rareness of the *M*-values is expressed as a logarithm of the possibility.

The *M* scores for SMB gene cluster candidates can be distinguished from other VCs when the values are sufficiently large, but other VCs still yield many noisy peaks (Figure 3, right-middle). Therefore, we tested several equations to reduce the signal-to-noise ratio of the *M* scores for SMB cluster candidates, yielding the final form of the MIDDAS-M equation:

(2) $$\omega_{i,\;ncl}=-{\left(\frac{M_{i,\;ncl}-\bar{M_{ncl}}}{\sigma_{M,\;ncl}}\right)}^{d}\log P_{i,\;ncl}$$

where *M_ncl_* and σ*_M,ncl_* are the mean and the standard deviation, respectively, of all *M* scores at a given cluster size *ncl, d* is a positive odd integer representing the order of the moment (set to 3 as default), and *P_i,ncl_* is the occurrence probability of *M_i,ncl_* in the distribution of all *M* scores at *ncl*. In Equation (2), two components result in the high sensitivity of MIDDAS-M detection: the moment and the rareness of the *M* scores (Figure 4). The moment, described as a *Z*-scored *M*-value to the *d*th power, indicates the degree of deviation from the normal distribution, whereas the rareness is described as the logarithm of the *M* score probability multiplied by −1, drastically emphasizing values with low probability, which will be closer to zero. The high sensitivity of MIDDAS-M is mainly derived from the moment, but the rareness also contributes to the sensitivity, especially when the template genome sequence is not well-defined. The moment can also be described as the degree of unbalance in a distribution. Gene induction ratios are normally distributed and symmetrical with respect to the *y*-axis, but the summed induction ratios, *M*, are unevenly distributed away from the normal distribution when the functional SMB gene cluster(s) are included in the analyzed transcriptome data. Most *M* values in the bell-shaped distribution are near zero because of the normalization, whereas the *M* scores of possible SMB VCs become larger than 1; subsequently raising the *M* scores to the power of integer *d* yields quite large numbers.

**Figure 5:**
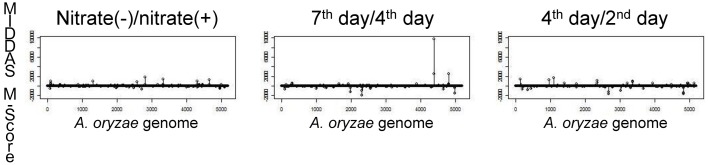
**Results of MIDDAS-M analysis of the KA biosynthesis gene cluster**. MIDDAS-M scores were calculated for the entire *A. oryzae* genome using three pairs of conditions as in Table 3.

Figure 5 shows the results of MIDDAS-M analysis using the same dataset as in Table 3 to detect the KA biosynthesis gene cluster. A strong peak in the MIDDAS-M scores was obtained using only the data from the 7th day/4th day pair but not from the 4th day/2nd day pair or the nitrate(−)/nitrate(+) pair. These results are consistent with our previous results that the 7th day/4th day pair yielded more accurate gene rankings. However, statistical evaluation using MIDDAS-M correctly identified the KA biosynthesis genes even when datasets from the undesirable conditions were included.

## Comparison of MIDDAS-M with SMURF and antiSMASH

**Figure 6:**
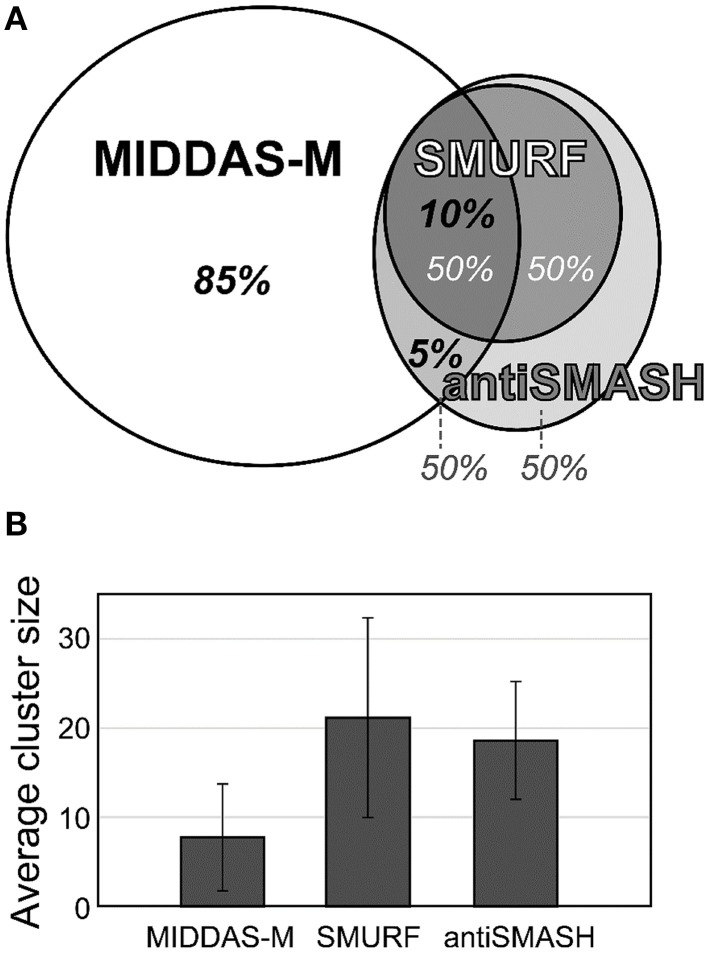
**Comparison of gene clusters in *A. flavus* detected by MIDDAS-M, SMURF, and antiSMASH**. **(A)** Among the candidate SMB gene clusters detected by MIDDAS-M, 10% and 5% were also predicted by SMURF and antiSMASH, respectively. Conversely, approximately half the clusters predicted by SMURF or antiSMASH were detected by MIDDAS-M. **(B)** The average cluster size of VCs detected by MIDDAS-M SMURF and antiSMASH.

The most valuable feature of MIDDAS-M analysis is that, unlike other tools for SMB gene cluster detection, it detects functional SMB gene clusters independent of the known motif sequences of the SMB core genes such as PKS, NPRS, and terpene cyclase (TC). Table 4 summarizes the number of SMB gene cluster candidates in *Aspergillus flavus* and *Fusarium verticillioides* detected by MIDDAS-M compared to the number detected by SMURF (Khaldi et al., 2010) and antiSMASH (Medema et al., 2011; Blin et al., 2013), which are the major tools that use motif information from the SMB core genes. The total number of clusters detected by MIDDAS-M, SMURF, or antiSMASH differs between *A. flavus* and *F. verticillioides*; the number of clusters detected by MIDDAS-M or SMURF/antiSMASH is >5-fold or ~2-fold higher, respectively, in *A. flavus* than in *F. verticillioides*. This difference may be a result of differences in the variety and sizes of the datasets used for the two species: for *A. flavus*, 28 datasets were obtained under various culture conditions, including liquid and maize solid media, whereas for *F. verticillioides*, only 4 datasets were obtained as time series using the same liquid medium. In *A. flavus*, 55 clusters were detected by SMURF, and 76 clusters were detected by antiSMASH; 49% and 46% of these clusters were also detected by MIDDAS-M, respectively. The number of clusters detected by MIDDAS-M are lower in *F. verticillioides*, but the percentage of SMURF (24%) and antiSMASH (27%) clusters detected by MIDDAS-M were similar, as in *A. flavus*. These data suggest that a certain percentage of the SMB gene clusters containing core genes are functional, whereas the rest might contain pseudo or silent genes that lost function or are inactive under most conditions. The percentage relationship between the genes detected by MIDDAS-M, SMURF and/or antiSMASH is presented as a Venn diagram for *A. flavus* (Figure 6A). Among the VCs detected by MIDDAS-M, 10% and 15% were also detected by SMURF and antiSMASH, respectively; the remaining 85% possess no known SMB core genes. Many of the VCs detected by MIDDAS-M may still be candidates for novel SMB gene clusters, as many contain “keystone” genes involved in secondary metabolism, such as P450, MFS transporters, and C6 transcription factors, and the occupancy increases according to the ω score (Umemura et al., 2013a).

**Table 4:** **The number and characteristics of the SMB gene cluster candidates detected by MIDDAS-M**.

**Strain**	MIDDAS-M^*1^	**SMURF**	By MIDDAS-M^*2^	**%**	**antiSMASH**	By MIDDAS-M^*2^	**%**
*A. flavus*	240	55	27	49	76	35	46
*F. verticillioides*	47	29	7	24	30	8	27

*1 *The threshold is ≥1016.7 for A. flavus and ≥499.4 for F. verticillioides*.

*2 *Counted when one or more genes in the cluster are detected by MIDDAS-M*.

**Figure 7:**
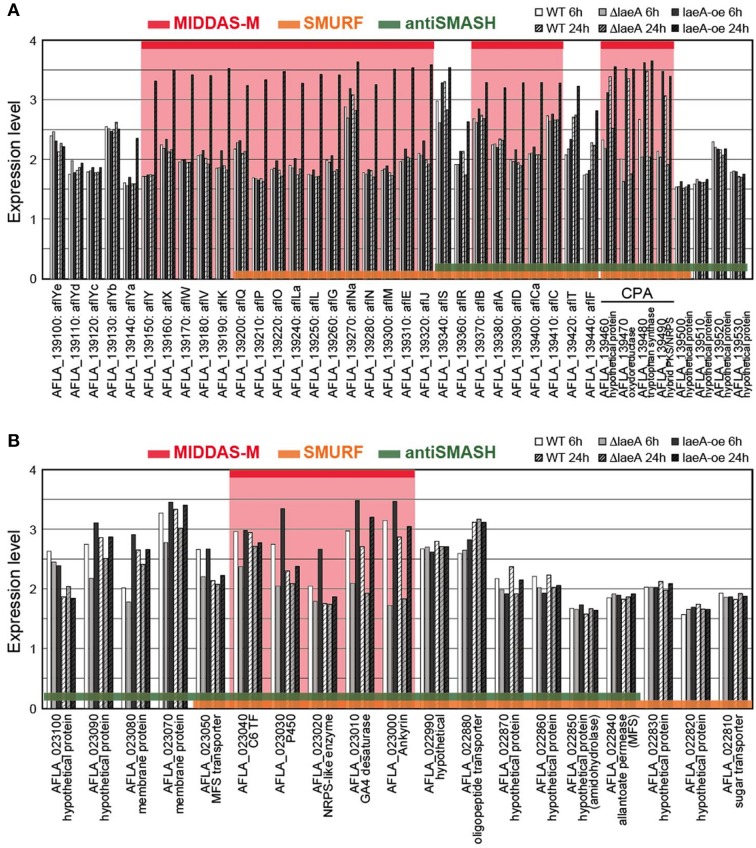
**Comparison of expression levels of gene clusters commonly detected by MIDDAS-M, SMURF, and antiSMASH**. Transcriptional expression levels of the genes in the chromosomal regions containing **(A)** the aflatoxin and cyclopiazonic acid biosynthetic gene clusters and **(B)** another unknown gene cluster containing an SMB core gene AFLA_023020, which were commonly detected by MIDDAS-M, SMURF, and antiSMASH, are indicated in a logarithmic scale. The values were obtained from a control strain and a deletion and an over-expression mutants at 6 h and 24 h in the transcriptome dataset of GSE15435 in the Gene Expression Omnibus database (http://www.ncbi.nlm.nih.gov/geo/). Gene IDs are indicated together with corresponding annotations from the NCBI database.

The cluster sizes predicted by MIDDAS-M are often smaller than those by SMURF and/or antiSMASH (Figure 6B). Of the VCs detected by MIDDAS-M, those composed of three genes are frequently observed (>60% for *A. flavus*), whereas most clusters predicted by SMURF or antiSMASH harbored 10~20 genes (Appendix S2) (Umemura et al., 2013a). The average cluster size detected by MIDDAS-M is 4.1, whereas those by SMURF and antiSMASH are 13.1 and 15.3, respectively, in *A. flavus* (Figure 6B). Frequency of the small gene clusters detected by MIDDAS-M is obviously higher than when assuming random distribution of the genes on the genome. Further, smaller size of the MIDDAS-M gene clusters does not depend on the absence of SMB core genes. Figure 7 shows expression of genes in two clusters detected by all the three methods but with significantly shorter length by MIDDAS-M than by the others as examples. MIDDAS-M predicted the aflatoxin biosynthesis gene cluster as two separate clusters (AFLA_139150–AFLA_139320 and AFLA_139370–AFLA_139410), which lacks five genes less than its actual number in total (Figure 7A) (Yu et al., 2004). The genes essential for the aflatoxin biosynthesis, AFLA_139140 (*aflYa*), AFLA_139340 (*aflS*), AFLA_139360 (*aflR*), AFLA_139420 (*aflT*), and AFLA_139440 (*aflF*) were excluded because their induction levels were significantly lower than those detected as the clusters. On the other hand, MIDDAS-M predicted the aflatoxin and cyclopiazonic acid (Chang et al., 2009) biosynthesis gene clusters separately, whereas SMURF and antiSMASH predicted the two clusters combined into a single cluster. Similarly, MIDDAS-M predicted another cluster, AFLA_023000–AFLA_023040, significantly shorter than in the case of SMURF and antiSMASH (Figure 7B). When judged directly from the expression profiles, AFLA_022810–AFLA_022990, which exhibit almost no expression differences in any conditions used in the analysis, can be apparently excluded. However, AFLA_023050–AFLA_023100, which seem to have induction/repression similar to but weaker than the genes in the cluster might be included in the cluster. The two examples above strongly suggest that MIDDAS-M can be fine-tuned (introduction of optional calculation for example) to make its prediction more accurate and practical by taking various experimentally validated results into consideration.

Table 5 shows the percentages of clusters containing a C6 transcription factor, an MFS transporter and a P450 enzyme in relation to the three detection methods. Of the gene clusters predicted by MIDDAS-M, SMURF and antiSMASH, 4%, 23%, and 17% contained a C6 transcription factor, respectively. The significantly smaller value for MIDDAS-M is thought to be due to smaller induction ratio of the transcription factor than that of the other genes in the clusters in general. The percentage of the C6 transcription factor further decreased to 2% when the clusters detected commonly by MIDDAS-M and SMURF/antiSMASH were used. However, because the value was calculated from only a small subset of the entire clusters (approximately 15% of the clusters detected by MIDDAS-M), it is not clear if the decrease might have any biological meaning. Smaller percentage of the clusters containing an MFS transporter detected by MIDDAS-M is thought to be due to smaller induction ratio of MFS transporters in general, similarly to the case of *aflT*, the transporter gene in the aflatoxin gene cluster (Figure 7B).

**Table 5:** **The percentage of clusters containing a C6 transcription factor, an MFS transporter, and a P450 in *A. flavus***.

**Detection method**	**C6 (%)**	**MFS (%)**	**P450 (%)**
MIDDAS-M	4	10	9
SMURF	23	36	36
antiSMASH	17	28	34
MIDDAS-M and SMURF/antiSMASH	2	7	30
MIDDAS-M only	5	10	5

**Figure 8:**
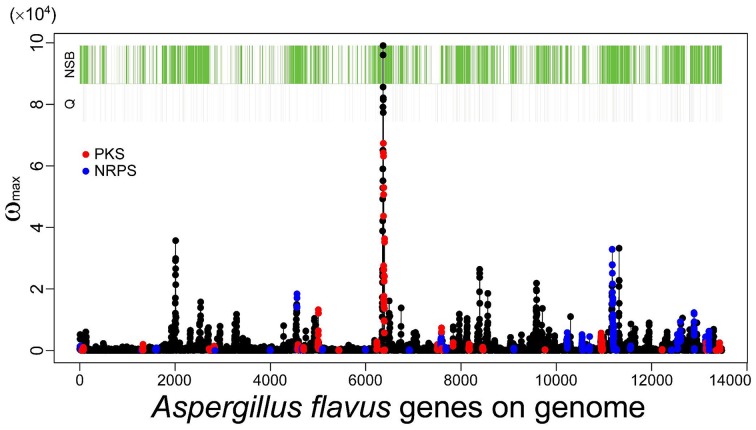
**MIDDAS-M peaks in *A. flavus* with indicators for the NSB regions, Q-genes, and PKS/NRPS gene clusters predicted by SMURF**. The red and blue marks overlaid on the MIDDAS-M peaks indicate the genes predicted as PKS and NRPS, respectively, by SMURF. The green and gray bars at the top of the figure indicate the genes in NSBs identified from the comparison between the *A. flavus* and *A. nidulans* genomes and the Q-genes in the KOG database, respectively.

As described in the Introduction, genes belonging to category Q in the KOG classification, secondary metabolic genes, tend to localize to NSBs regardless of their distance from the telomeres or their expression levels (Umemura et al., 2012). SMB genes detected by MIDDAS-M also exhibit localization to NSBs: 72% of the identified genes are located in NSBs in *A. flavus*, even though NSBs comprise only 29% of the genome compared with the *A. nidulans* genome (Umemura et al., 2013a). Figure 8 shows a projected view of all MIDDAS-M peaks obtained using transcriptome data from 28 culture conditions (previously presented in a 3D view) (Umemura et al., 2013a), with indicators for NSB regions, KOG Q-genes, and PKS/NRPS gene clusters predicted by SMURF. The localization of MIDDAS-M-detected clusters to NSBs is more obvious in the 3D view in our previous report (Umemura et al., 2013a).

There are several reports of global regulators for fungal secondary metabolism such as VeA and LaeA (Keller and Hohn, 1997; Reyes-Dominguez et al., 2010; Soukup et al., 2012; Bok et al., 2013). By comparing the *laeA* over-expression and its deletion mutants of *A. flavus*, 10 candidate clusters including those for aflatoxin and cyclopiazonic acid are detected by MIDDAS-M. This detection number is comparable to the number (17 clusters) obtained from comparison between *A. flavus* and *A. oryzae* under maize culture. Similarly, approximately 10 candidate clusters are detected by comparing transcriptomes from different temperatures such as 28°C/37°C under liquid A&M and solid maize cultures (Georgianna et al., 2010).

## Toward comprehensive analysis of SMB gene clusters

**Figure 9:**
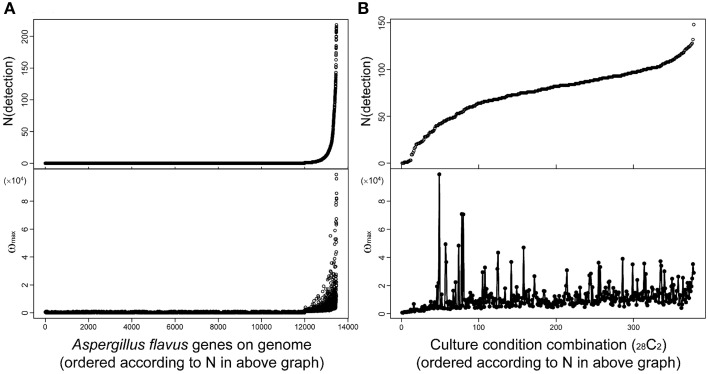
**Relationships between height and number of detected MIDDAS-M peaks in *A. flavus***. Top: The number of peaks detected by MIDDAS-M from 28 datasets of transcriptome data for *A. flavus*, according to the genes **(A)** and the culture condition combinations (CCC; _28_*C*_2_ = 378) **(B)**. The numbers are sorted in ascending order. Bottom: The maximum ω score for each gene **(A)** and CCC **(B)**.

Fungal secondary metabolites tend to be produced under limited culture conditions. Therefore, comprehensive analysis of transcriptome data obtained under various culture conditions is essential for the discovery of novel SMB gene clusters. MIDDAS-M is suitable for this purpose because it can concurrently process large-scale transcriptome datasets. For example, we used 28 transcriptome datasets obtained under various liquid and solid media culture conditions for our MIDDAS-M analysis of *A. flavus* and several datasets for our analysis of *A. oryzae*, resulting in 378 sets of gene induction ratios (Umemura et al., 2013a). It is not difficult to perform MIDDAS-M analysis of 100 or more transcriptome datasets using recent high performance computers. Figure 9 shows the relationship between the number and height of the detected MIDDAS-M peaks for each gene (Figure 9A) and for each culture condition combination (CCC) (Figure 9B). For each gene, the detected number of CCCs increases exponentially and strongly correlates with the peak value; that is, when a gene is detected as a member of a candidate VC in various CCCs, its maximum peak value tends to be strong (Figure 9A). SMB gene clusters that are rarely expressed tend to show weaker MIDDAS-M peaks; therefore, it is better to survey relatively small peaks to identify rare SMB gene clusters, which have a high probability of being novel.

Plotting the number of detected genes vs. CCCs after sorting by the number of detected genes yields an *S*-curve (Figure 9B, top), while the curve from plotting the number of detected genes per gene is very different (Figure 9A, top). One of the reasons is that the CCC data includes all possible combinations of two datasets from 28 conditions, so that one dataset appears 27 times, yielding a total of 378 CCCs. The number of detected peaks per CCC does not correlate with the maximum ω-value, but the highest peaks were observed for the cultivation condition pairs harboring a relatively small number of detected peaks. This effect may be partially derived from the principle of MIDDAS-M itself; that is, the ω-value has relation to the number of genes detected because the ω-value is evaluated as the degree of deviation from the normal distribution. When many VCs deviate from the normal distribution, the statistical center of the distribution may shift closer to the data points with large deviations, resulting in underestimated deviation values.

**Figure 10:**
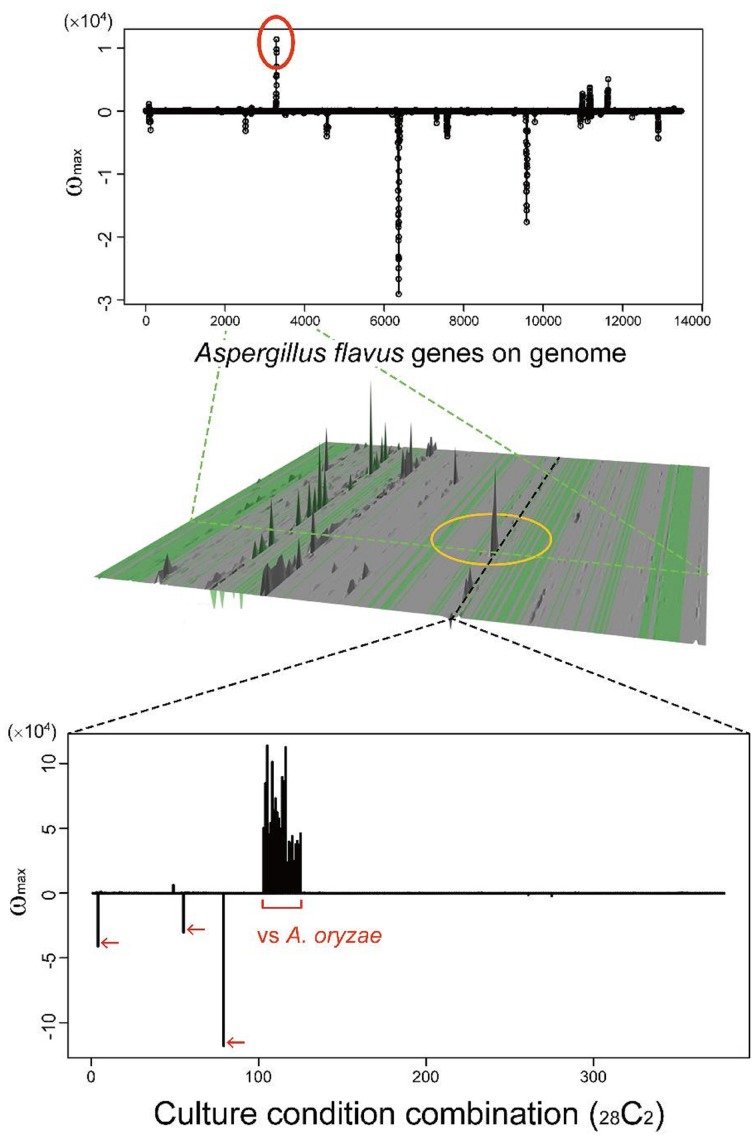
**A candidate SMB gene cluster rarely detected among various culture condition combinations**. The peak in the middle of the 3D figure (Umemura et al., 2013a) is detected only in limited culture condition combinations (CCCs); the graph cut-outs show the position of a corresponding CCC (above) and gene (below).

Many secondary metabolites are produced only under limited culture conditions. Several culture and media conditions are known to be suitable for the production of secondary metabolites, including solid maize culture, but the knowledge is empirical. This knowledge gap is one of the main reasons SMB genes have been difficult to identify. MIDDAS-M allows us to analyze a large number of culture conditions simultaneously to identify conditions in which rare SMB gene cluster(s) are expressed. As a case study, we examined a rare cluster that was observed in limited CCCs, as described in our previous report (Umemura et al., 2013a). The cluster, designated by a yellow circle in the middle of Figure 10, is detected only by comparing *A. oryzae* to *A. flavus* datasets, i.e., the cluster is expressed only in the former species. *A. oryzae*, a close relative of *A. flavus*, rarely produces secondary metabolites compared to *A. flavus*, despite the large number of orthologs found between the two species (Payne et al., 2006). *A. oryzae* is used in the food industry because of this low production of secondary metabolites. The functional annotations of the genes in this cluster from NCBI indicate that it contains a hydrolase, a dehydrogenase and two neighboring genes annotated as P450 and MFS transporters, which are often found in SMB gene clusters. In contrast, significantly more possible SMB gene clusters were detected in *A. flavus* using MIDDAS-M as expected. Considering the remarkable differences between the two species in terms of SMB gene expression, the corresponding gene cluster in *A. flavus* might be inactivated due to the high mutation frequency in NSBs. Alternatively, the product of this cluster may be beneficial to *A. oryzae*, yielding a good flavor during fermentation for example. This result demonstrates that MIDDAS-M analysis can be used to find rare SMB gene clusters due to its comprehensive analysis capabilities.

Recently, we have developed another software tool, MIPS-CG (motif-independent prediction without core genes), for the identification of completely novel SMB gene clusters with no known core genes (Takeda et al., 2014). MIPS-CG first detects gene clusters by searching for a pair of clustered genes with significant similarity between two genomes without using any known sequences or motifs. More importantly, MIPS-CG selects potential SMB gene clusters from the gene cluster candidates based on the discovery that SMB gene clusters are highly enriched on NSBs (Machida et al., 2005). The localization of SMB gene clusters on NSBs are clearly conserved in the results by the projected view of MIDDAS-M as described above. By applying appropriate values for several parameters, MIPS-CG successfully detected most of the known SMB gene clusters (21/24 gene clusters) from 10 filamentous fungal genomes without large differences from the experimentally determined positions of the cluster boundaries. As the two software tools, MIDDAS-M and MIPS-CG, use principally different algorithms to detect SMB gene clusters, combining the two software tools is expected to increase the probability and accuracy of detecting novel SMB gene clusters, especially ones without any known core genes.

## Discovery of a novel type of fungal secondary metabolic pathway

**Figure 11:**
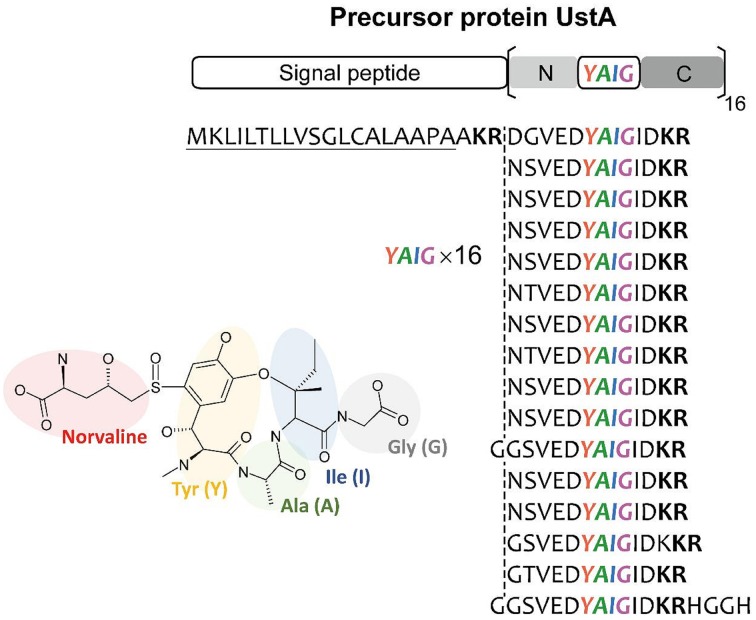
**The structure of ustiloxin B and its precursor protein, UstA**. Ustiloxin B consists of a cyclic tetrapeptide, Tyr-Ala-Ile-Gly (YAIG), whose tyrosine is modified with the non-proteinogenic amino acid norvaline (Umemura et al., 2014). The cyclic YAIG amino acid sequence is directly encoded in the gene for the precursor protein UstA.

It is thought that there is virtually little example of SMB gene clusters without known SMB core genes discovered using bioinformatics before experimental detection. Nonetheless, MIDDAS-M successively detected the ustiloxin biosynthetic gene cluster, which does not include PKS, NRPS, or any other known SMB core genes. Successive detailed analyses of the gene cluster revealed that ustiloxin biosynthesis is the first example of a ribosomal biosynthetic pathway in filamentous fungi (Umemura et al., 2013a, 2014). Ustiloxin B is a cyclic tetrapeptide, Tyr-Ala-Ile-Gly, whose tyrosine is modified with a non-protein coding amino acid, norvaline (Figure 11). The structure first indicated that the compound is synthesized by NRPS, but none of the NRPS-specific catalytic domains, A, C, PCP, and TE, were included in the gene cluster or within 10 genes adjacent to it. Instead, we found a gene whose translated amino acid sequence contains a 16× repeat of short peptides, including “YAIG,” the exact sequence of the ustiloxin B cyclic moiety (Figure 11). Therefore, ustiloxin B is not synthesized by NRPS but is instead synthesized ribosomally as a precursor protein, UstA, followed by processing via peptidases (Umemura et al., 2014). Ribosomally synthesized natural compounds are designated as ribosomal peptides and have been reported in bacteria, plant, and cone snails, especially since the development of genome sequencing technologies in the twenty-first century (Velasquez and Van Der Donk, 2011; Schmidt, 2012; Arnison et al., 2013; Yang and Van Der Donk, 2013). Except one example, amanitin, produced by the *Amanita* mushroom (Hallen et al., 2007), the synthesis of ribosomal peptides in filamentous fungi had not been reported until the identification of the ustiloxin B biosynthetic gene cluster. The highly repeated structure of the precursor protein, UstA, has not been observed in bacteria, thus it might be a characteristic feature specific to filamentous fungi. By searching biosynthetic pathways similar to the ustiloxin one in filamentous fungi by analyzing their genome sequences and metabolite profiles, the world of fungal secondary metabolites will be widely extended.

Ustiloxin B, an inhibitor of microtubule assembly, was first discovered as a metabolite of the fungus *Ustilaginoidea virens*, which is a rice pathogen that causes false smut disease. As the compound was not known to be produced by *A. flavus* until the identification of the gene cluster by MIDDAS-M, the ustiloxin B biosynthetic gene cluster was discovered using a “gene to compound” strategy. This strategy includes certain inevitable difficulties. The production of secondary metabolites is generally unstable and often reduced in laboratory environments; thus, it is extremely difficult to identify target compounds among the thousands of other metabolites produced. Other experimental limitations include the detection sensitivity limit of the available instruments, the absence of a suitable extraction solvent, and the rapid degradation or volatile characteristics of the compound. We experienced all these situations while preparing fifty or more deletion mutants based on MIDDAS-M prediction, and we learned that at least the potential presence of a compound of interest should be indicated in the culture conditions. The transcriptome-based methodology adopted by recent tools is the most straightforward and fastest approach for a “compound to gene” strategy because it detects only the expressed and potentially functional clusters in the culture conditions suited for the production of the compound.

## Conclusion

Genome sequencing has shed light on comprehensive analysis of SMB gene clusters in filamentous fungi. In contrast to the importance of SMB gene clusters with the core genes such as PKS, NRPS, DMAT, and TC, lack of potential methodologies has made exploring SMB gene clusters without the core genes extremely difficult. MIDDAS-M is a powerful method for detecting novel SMB gene clusters, especially functionally active ones. Although the detected clusters were frequently located in NSBs, where SMB genes are highly enriched, only a few examples currently available confirm that the newly identified clusters actually produce secondary metabolites. Following points should be considered when using MIDDAS-M to search for SMB gene clusters: (i) the detected clusters may not be SMB gene clusters but instead have a currently unknown function; (ii) the clusters may not produce active polypeptide (enzymes, transporters, transcription factors, and so on); or iii) the products may be active yet remain inactive in the overall metabolism (possibly because part of the cluster is missing). Thus, detailed analyses of the novel clusters detected by MIDDAS-M are necessary to characterize the novel clusters and extend our knowledge of novel secondary metabolism.

The dramatic acceleration in sequencing fungal genomes due to the development of sequencing technologies after the twenty-first century (Chiang et al., 2009; Lim et al., 2012) allows the comprehensive analysis of SMB gene clusters from entire fugal species. Thus, the “gene to compound” strategy is quite attractive despite the difficulties described in the end of the previous section. This strategy is applied to recent attempts to awaken cryptic or silent SMB gene clusters in fungi by manipulating regulatory genes. In spite of dependence of MIDDAS-M on gene expression, MIPS-CG predicts SMB gene clusters only from genome sequence information. Combining these methods and motif-dependent methods such as SMURF and antiSMASH should significantly accelerate comprehensive analysis of secondary metabolism of filamentous fungi.

### Conflict of interest statement

The authors declare that the research was conducted in the absence of any commercial or financial relationships that could be construed as a potential conflict of interest.
